# White Matter Integrity Abnormalities in Healthy Overweight Individuals Revealed by Whole Brain Meta-Analysis of Diffusion Tensor Imaging Studies

**DOI:** 10.1155/2023/7966540

**Published:** 2023-10-23

**Authors:** Xiaodong Cheng, Wenchang Wang, Chen Sun, Yana Sun, Cong Zhou

**Affiliations:** ^1^Department of Psychiatry, Shandong Daizhuang Hospital, Jining, China; ^2^School of Mental Health, Jining Medical University, Jining, China; ^3^Department of Nutrition, Affiliated Hospital of Jining Medical University, Jining, China; ^4^Department of Psychology, Affiliated Hospital of Jining Medical University, Jining, China

## Abstract

**Objective:**

This study aimed to conduct a coordinate-based meta-analysis (CBMA) to investigate white matter (WM) abnormalities in healthy individuals with overweight or obesity.

**Methods:**

A systematic literature search using Web of Science and PubMed datasets was performed. Original investigations that used diffusion tensor imaging (DTI) to explore fractional anisotropy (FA) differences between healthy overweight/obese individuals and normal weight controls were collected. The meta-analysis was conducted using the seed-based *d* mapping (SDM) software, employing stringent thresholds for significance. Sensitivity analyses and meta-regression analysis were also performed to examine the robustness of the results and explore potential associations with age and body mass index (BMI).

**Results:**

The analysis included five studies comprising 232 overweight/obese individuals and 219 healthy normal weight controls. The findings showed that overweight/obese individuals exhibited reduced fractional anisotropy (FA) in specific regions, namely, the right superior longitudinal fasciculus (SLF), the splenium of the corpus callosum (CC), and the right median network, cingulum. Meta-regression analysis further revealed that these FA reductions were associated with age.

**Conclusion:**

These findings provided insights into the potential impact of overweight/obesity on cognition, emotion, and neural functions and highlighted the significance of early prevention and intervention for overweight on the basis of neuroimaging.

## 1. Introduction

Overweight as well as obesity is closely associated with 21 diseases, including cardiovascular, metabolic, digestive, respiratory, neurological, musculoskeletal, and infectious diseases [1, 2]. Moreover, there is a dose-response relationship between the degree of obesity and the risk of developing complex comorbidities. The higher the degree of obesity, the greater the risk of developing complex comorbidities [1]. Specially, obesity is related with increased risk of accelerated cognitive decline and emotional distress [3, 4], suggesting neurobiological changes.

One recent meta-analysis of structural MRI in obesity exhibited abnormalities in gray matter volume (GMV) in regional brain regions such as the frontal lobe, temporal lobe, precentral gyrus, and occipital lobe [5]. The study by Caunca and colleagues indicated that greater BMI and waist circumference were significantly associated with thinner cortices, particularly in individuals aged less than 65 years. This suggested that obesity might also be linked to alterations in cortical thickness, which is another key aspect of brain structure [6]. Diffusion tensor imaging (DTI) is a well-established MRI technique that has been extensively used to explore white matter (WM) properties at a microstructural level [7]. Previous neuroimaging evidence indicated that elevated body mass index (BMI) is also associated with reduced white matter integrity [8, 9]. Some portion of the corpus callosum (CC) and other vital neural tracts, such as the cingulate bundle, the superior longitudinal fasciculus (SLF), the inferior longitudinal fasciculus (ILF), and the uncinate fasciculus (UF), are particularly susceptible to the effects of body weight [10–12]. This could potentially contribute to impairments in reward-related behavior, cognitive and inhibitory control, and memory and decision-making processes among individuals who are overweight or obese. One systematic review [11] examined 31 cross-sectional studies comparing individuals with obesity to normal weight controls. The majority of these studies reported decreased fractional anisotropy (FA) and increased mean diffusivity (MD) in WM, suggesting altered properties. However, the pattern of alterations varied across studies. The inconsistency in findings may be attributed to the substantial heterogeneity among the cohorts included in the study.

Meta-analysis is a valuable technique that allows researchers to consolidate and analyze data from multiple studies, providing a more robust and applicable estimate of the effect size. By combining data from various sources, meta-analysis increases the sample size and statistical power, making it easier to detect even small effects and reducing the risk of erroneous findings, such as false positives or false negatives [13]. Coordinate-based meta-analysis (CBMA) has gained prominence as a method to address the discrepancies observed in various studies exploring neuroimaging results. One recent CBMA aimed to identify consistent white matter alterations in obesity [10]. The researchers performed a meta-analysis using FA datasets and found a reduction in FA in the genu of the CC in individuals with obesity. This finding was further validated using an independent sample, supporting the association between obesity and reduced WM integrity in this specific brain region involved in executive function. The study highlighted the need for further research on the mechanisms linking obesity and white matter integrity loss. A lately published CBMA [12] found reduced FA in the genu and splenium of the CC, middle cerebellar peduncles, anterior thalamic radiation, corticospinal projections, and cerebellum. The region of interest meta-analysis replicated the associations between obesity and lower FA in the genu and splenium of the CC and middle cerebellar peduncles. The effect size of obesity-related brain changes was small to medium. The study suggested that obesity-related WM alterations were localized rather than diffuse, and understanding these brain correlates may help identify risk factors and targets for prevention or treatment.

The aforementioned meta-analysis considered obese cohorts with comorbidities such as type 2 diabetes (T2DM), but there is a lack of research specifically examining WM characteristics in healthy individuals who are overweight or obese. Herein, we intended to conduct a CBMA solely on healthy overweight or obese individuals compared with healthy individuals with a normal weight. Building on previous findings from published articles, our hypothesis was that the overweight/obese group would exhibit alterations in WM microstructures, particularly in central nerve tracts such as the SLF and the CC.

## 2. Methods

### 2.1. Literature Search Strategy

We registered our CBMA protocol with PROSPERO (https://www.crd.york.ac.uk/PROSPERO) under registration number CRD42022303123 and adhered to PRISMA guidelines [14–16] in conducting this meta-analysis. We conducted a systematic search of Web of Science and PubMed datasets for peer-reviewed articles published until February 28, 2023, using advanced search functions and the following keywords: (“obesity” or “obese” or “overweight” or “body mass index” or “waist circumference” or “waist-to-hip ratio”) and ((“diffusion tensor imaging” or “DTI” or “diffusion magnetic resonance imaging”) or (“fractional anisotropy”) or (“tract‐based spatial statistics” or “TBSS”)).

### 2.2. Study Selection

We included only English-language original investigations published in peer-reviewed journals that used the whole brain approach to explore FA differences between healthy overweight/obese individuals and healthy normal weight controls, reporting their results in Montreal Neurological Institute (MNI) or Talairach coordinates and using a threshold for significance. We excluded case reports, reviews, meta-analyses, and tractography-based only studies, as well as studies with no direct between-group comparison or from which peak coordinates or parametric maps could not be obtained.

### 2.3. Quality Assessment and Data Extraction

Following the guidelines for neuroimaging meta-analyses [17], two authors (XC and WW) independently searched the literature, assessed the quality of the retrieved articles, and extracted and cross-checked data from eligible articles. Both authors also separately checked the quality of the final studies. For each study, we recorded the first author, cohort size, demographics (gender and age), BMI, and data for SDM calculations, including the coordinates of main findings and values related to effect size (*Z*-statistics, *t*-statistics, and *P* value).

### 2.4. SDM Meta-Analysis

The SDM software (https://www.sdmproject.com) [18, 19] was utilized to conduct a comprehensive meta-analysis of regional FA differences between individuals classified as healthy overweight/obese and those of normal weight. The standardized SDM tutorial and previous meta-analysis studies were referred during the analysis. In brief, the SDM technique employs effect sizes in conjunction with peak coordinates extracted from databases of statistical parametric maps to generate maps depicting the original effect size of FA differences between healthy overweight/obese individuals and healthy normal weight controls [20]. We applied more stringent thresholds including *P* < 0.001, a peak height threshold of *Z* = 1.00, and a cluster size threshold of 10 voxels.

### 2.5. Jackknife Sensitivity Analyses

To examine the robustness of our findings, we performed a jackknife sensitivity analysis. This approach entailed repeating the primary analysis for a total of *n* times (where *n* corresponds to the number of datasets included) and systematically excluding one study at a time. We considered a brain region highly reliable if it remained significant after performing jackknife sensitivity in all or most of the combinations of studies [18].

### 2.6. Meta-Regression Analysis

To minimize the detection of potential correlations between demographics and neuroimaging, a more stringent threshold of *P* < 0.0005 was employed. This threshold was consistent with previous meta-analyses and the recommendations of the SDM creators [18]. We explored the associations between FA alterations and mean age and BMI. The aforementioned procedures were only implemented in brain regions identified in the main analysis.

## 3. Results

### 3.1. Characteristics of the Included Studies

**Figure 1:**
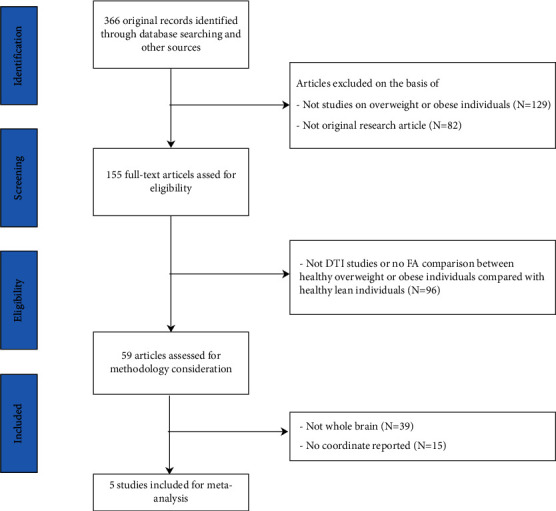
Flow diagram of study selection process.

A total of 366 articles were initially identified. Based on the inclusion and exclusion criteria, a total of 5 articles were finally included [21–25]. The final sample comprised 232 overweight individuals and 219 healthy controls. The basic characteristics of the included studies are shown in Table 1, and the study selection processes are presented in Figure 1.

### 3.2. Regional FA Differences in Overweight/Obese Individuals

**Figure 2:**
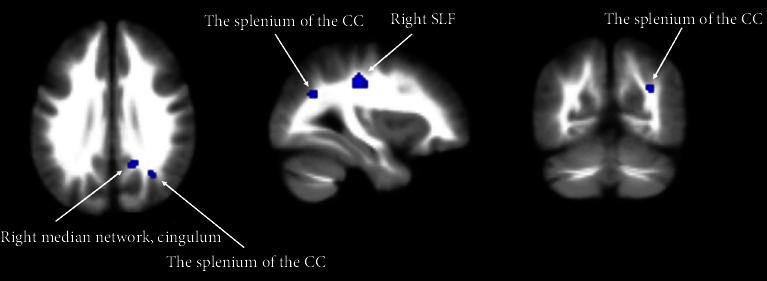
Decreased FA in healthy overweight/obese individuals in axial, sagittal, and coronal views. Significant cluster is overlaid on MRIcron template for Windows for display purposes only.

Overweight/obese individuals exhibited FA reductions in the right superior longitudinal fasciculus (SLF), the splenium of the corpus callosum (CC), and the right median network, cingulum (Figure 2 and Table 2). There were no observed increases in FA. The whole brain jackknife approach confirmed the reliability of our findings as the decreased FA values persisted across all combinations (Table 2).

### 3.3. Meta-Regression Analysis

Our meta-regression analysis indicated negative correlations between age and FA in all above three clusters, with statistical significance defined by the strict threshold of *P* < 0.0005 (Table 3). That is, with age increasing, the FA value of these regions would get lower.

## 4. Discussion

Our study aimed to investigate WM abnormalities in healthy individuals with overweight/obesity through conducting a CBMA. The findings revealed that overweight/obese individuals exhibited reduced FA in specific regions, including the right SLF, the splenium of the CC, and the right median network, cingulum. Importantly, meta-regression analysis further revealed that these FA reductions showed a significant association with age, suggesting age-related changes in these WM regions among overweight/obese individuals. The findings provided insights into the potential impact of overweight on cognition, emotion, and neural system functions. Additionally, this study can contribute to supplementing more specific evidence to drive the development of prevention and intervention strategies for overweight/obesity-related brain structural changes and might serve as an early warning sign for cognitive disorders associated with obesity and provide guidance for relevant treatments and rehabilitation.

Consistent with previous studies [10, 12], our results indicated that overweight/obesity is associated with decreased FA in vital neural bundles. The SLF plays a crucial role in connecting the frontal and parietal lobes, which are involved in executive functions such as attention and working memory [26]. The CC is responsible for interhemispheric communication, and alterations in its microstructure have been linked to cognitive deficits and emotional distress [10]. The cingulum connects various brain regions involved in emotion regulation and has been implicated in mood disorders [27]. Therefore, the observed FA reductions in these regions might be related to cognitive and emotional impairments commonly observed in overweight/obese individuals. Besides, the aberrant WM microstructure within these brain regions might be responsible for individuals' abnormal eating behavior and could also be a consequence of overweight. Our meta-regression analysis further revealed a negative correlation between age and FA in the identified brain regions. This was consistent with previous research suggesting that older age was associated with decreased FA in various brain regions [28] and also in accord with the structural MRI findings that brain microstructures of obese individuals might further deteriorate with aging [6]. The relationship between age and FA in overweight/obese individuals may be a result of the cumulative effects of obesity-related factors such as chronic inflammation and oxidative stress, which accelerate brain aging processes [29]. However, more research is needed to elucidate the underlying mechanisms and explore potential interactions between obesity, age, and brain white matter integrity.

Limitations of our study include the relatively small number of included studies and heterogeneity in participant characteristics, such as differences in age, sex, and BMI. Future studies should address these limitations by including larger sample sizes and controlling for potential confounding factors. Additionally, longitudinal studies are needed to determine the temporal relationship between obesity and alterations in brain WM microstructure. Furthermore, additional DTI parameters such as MD, axial diffusivity (AD), and radial diffusivity (RD) would offer valuable insights into characterizing microstructural WM alterations in healthy overweight individuals. Regrettably, we encountered limitations in conducting meta-analyses involving these DTI metrics due to the inadequacy of available datasets. Lastly, we only utilized peak coordinates and effect sizes from published studies, rather than the original statistical brain maps, which might result in partial loss of original information.

## 5. Conclusion

In conclusion, our study found that healthy overweight/obese individuals exhibited age-related decreased FA in the right SLF, the splenium of the CC, and the right median network, cingulum. These findings provide valuable insights into the potential impact of overweight/obesity on cognition, emotion, and neural system functions. The study also highlights the importance of developing prevention and intervention strategies for brain structural changes associated with overweight/obesity and suggests that these changes may serve as early warning signs for cognitive disorders in this population.

## Figures and Tables

**Table 1:** Characteristics, scanning methods, and neuroimaging alterations of the participants in the 5 studies included in the meta-analysis.

Study	Subjects, *n* (female, *n*)		Age, years		BMI (kg/m^2^)	Scanner (T)	Diffusion encoding orientations	*b*value (s/mm^2^)	Scan time	DTI voxel size (mm^3^)	Neuroimaging alterations
	OW/OB	Lean	OW/OB	Lean							
Shott et al. [21]	18 (18)	24 (24)	28.7	27.4	34.8	3.0	25	1000	N/A	Thickness = 2.6 mm	Decreased FA observed in bilateral anterior corona radiata, superior corona radiata, sagittal stratum, and external capsule. ADC was greater in the right sagittal stratum and left superior corona radiata.
Papageorgiou et al. [22]	148 (77)	120 (76)	51.7	39.8	NA^a^	1.5	15	700	N/A	1 × 1 × 1	Decreased FA observed in superior longitudinal fasciculus R, corticospinal tract R, cingulum (cingulate gyrus) R, cingulum (hippocampus) R, white matter optic radiation R, and inferior longitudinal fasciculus R. No other diffusion index (MD, RD, or AD) difference was found.
Ottino-Gonzalez et al. [23]	31 (19)	21 (11)	31.1	29.9	30.8	3.0	30	1000	4 min 23 sec	Thickness = 2 mm	No differences were found in FA, MD, AD, and RD between groups.
Samara et al. [24]	18 (7)	41 (16)	29.8	29.5	35.7	3.0	23	1400	N/A	2 × 2 × 2	Decreased AD observed. No differences were found in FA, MD, and RD between groups.
Estella et al. [25]	17 (17)	13 (13)	38.0	34.7	33.6	3.0	30	1000	7 min	Thickness = 2 mm	No differences were found in FA, MD, AD, and RD between groups.

^a^The overweight group with BMI between 25.0 and 29.9 had 96 members; the obese group with BMI ≥30.0 had 52 members. AD: axial diffusivity; ADC: apparent diffusion coefficient; BMI: body mass index; FA: fractional anisotropy; L: left; MD: mean diffusivity; N/A: not available; OB, obese; OW, overweight; R: right; RD: radial diffusivity.

**Table 2:** FA reductions in healthy overweight individuals compared to lean controls revealed by the coordinate-based meta-analysis.

Regions	Maximum					Cluster	Jackknife sensitivity analysis
	MNI coordinates			SDM value	*P*	Number of voxels^*∗*^	
	*X*	*Y*	*Z*				
Right superior longitudinal fasciculus	30	−24	40	−1.914	0.000006497	98	5/5
Corpus callosum	32	−60	28	−1.719	0.000039101	46	5/5
Right median network, cingulum	16	−52	30	−1.696	0.000110865	37	5/5

^*∗*^All voxels with *P* < 0.001 uncorrected. FA: fractional anisotropy; MNI, Montreal Neurological Institute; SDM, seed‐based *d* mapping.

**Table 3:** Associations between FA alterations and age in healthy overweight individuals revealed by meta‐regression analyses.

Factor	Anatomic label	MNI coordinates			SDM value	*P*	Number of voxels
		*X*	*Y*	*Z*			
Age	Corpus callosum	32	−60	28	−2.302	0.000006497	26
	Right superior longitudinal fasciculus	28	−24	42	−2.264	0.000058711	25
	Right median network, cingulum	16	−52	30	−2.270	0.000058711	24

FA: fractional anisotropy; MNI, Montreal Neurological Institute; SDM, seed‐based *d* mapping.

## Data Availability

The dataset analyzed during the current study is available from the corresponding author on reasonable request.
